# The Double-Edge Sword of Natural Phenanthrenes in the Landscape of Tumorigenesis

**DOI:** 10.3390/molecules30061204

**Published:** 2025-03-07

**Authors:** Yan Liu, Ziwei Du, Chen Sheng, Guangshuai Zhang, Si Yan, Zhijun Zhang, Shuanglin Qin

**Affiliations:** 1 School of Pharmacy, Xianning Medical College, Hubei University of Science and Technology, Xianning 437100, China; liuyan20210915@163.com (Y.L.); duziwei0101@163.com (Z.D.); 18986401408@163.com (C.S.); zhanggs1999@163.com (G.Z.); yansi92674@163.com (S.Y.); 2 Research Center for Precision Medication of Chinese Medicine, FuRong Laboratory, Hunan University of Chinese Medicine, Changsha 410208, China

**Keywords:** natural plants, phenanthrenes, aristolochic acid, anti-tumor activity, toxicity-effect transformation

## Abstract

Phenanthrenes, which are polycyclic aromatic hydrocarbons comprising three benzene rings, exhibit a diverse range of functions. These compounds are utilized in the synthesis of resins, plant growth hormones, reducing dyes, tannins and other products. Notably, phenanthrenes possess significant pharmacological properties, including anti-tumor, anti-inflammatory and antioxidant activities, offering broad prospects for development, particularly in the fields of medicine and health. Interestingly, although aristolochic acid (AA) is a potent carcinogen, its lactam analogs can kill cancer cells and exhibit therapeutic effects against cancer. This provides a promising strategy for the toxicity-effect transformation of phenanthrenes. In this paper, we reviewed 137 articles to systematically review the anti-tumor potential and toxic effects of natural phenanthrenes isolated from the 19th century to the present, thus offering references and laying a foundation for their further research, development and utilization.

## 1. Introduction

**Figure 1:**
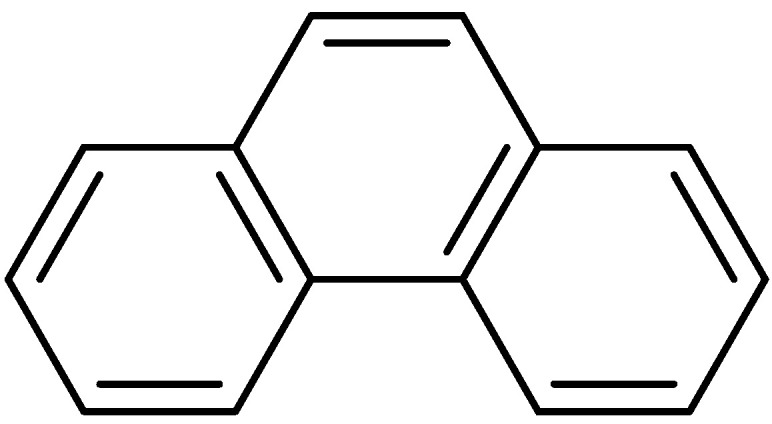
The parent structure of phenanthrenes.

Phenanthrenes are a type of aromatic hydrocarbon metabolite formed through the aromatic epoxidation coupling of styrene. Figure 1 illustrates the parent structure of phenanthrenes. Over the past decade, the incidence and mortality rates of malignant tumors in China have continued to rise. Currently, the 5-year relative survival rate for malignant tumors stands at approximately 40.5%. This represents an increase of about ten percentage points compared to ten years ago. In recent years, research into naturally occurring anticancer agents has gained popularity. The phenanthrenes reviewed are predominantly found in nature, with over 250 types identified, mostly oxygen-containing analogs derived from advanced plants. A substantial body of research indicates that the majority of phenanthrenes originate from higher plants, particularly within the Orchidaceae family, which includes 49 species such as Dendrobium Cymbidium, Elia, Jaws, Bletilla, Coelogyna, Cymbidium, Mayfly and Epidermis [1]. Phenanthrenes have also been identified in the *Dioscoreaceae*, *Asteraceae* and *Betulaceae* families. Typically, phenanthrenes are isolated from the entire plant, and research has also occasionally focused on the cortex, tubers or stems. In recent years, numerous studies have been conducted at home and abroad on the anti-tumor activity of phenanthrenes. For example, chrysotoxol and nudol extracted and isolated from *Dendrobium officinale* have inhibitory effects on liver cancer and osteosarcoma; the 7-methoxy-8-methyl-5-vinyl-9,10-dihydro-phenanthren-2-ol isolated from J.effususus showed inhibitory effects on A2780, A2780 cis, KCR, MCF-7, HeLa, HTB-26 and T47D human tumor cells; and the 5,5′,7,7′-tetramethoxy-9,9′,10,10′-tetrahydro-[3,3′-biphenanthrene]-2,2′-diol isolated from the orchid plant Panlongshen has moderate anticancer activity against liver cancer.

Although phenanthrenes themselves are not carcinogenic, they serve as prototype molecules for the biological studies of carcinogenic compounds such as benzo[a]anthracene, dibenzo[a,h]anthracene and benzo[a]pyrene. These compounds were classified as carcinogens by the World Health Organization in 2017 [2]. For instance, the metabolism of deuterated phenanthrene in humans has been used as a marker to assess the potential susceptibility of smokers to lung cancer [3]. Aristolochic acid, a nitrophenic acid compound, can cause various cancers such as urothelial, renal cell, hepatocellular, biliary tract and gastrointestinal cancers when ingested through Chinese herbal medicines, skin contact or the inhalation of herbal powder [4]. AAs comprise a group of nitrophenanthrene organic acids naturally present in Aristolochia plants such as *Radix Aristolochiae fangchi* and *Asarum caudigerellum* extensively utilized in traditional Chinese medicine as raw materials [5]. AAI, the most prevalent compound, is found in nearly all Aristolochia plants, often co-existing with aristolactams [4]. The primary toxic components in these compounds are AAI and AAII [6]. Catalyzed by nitroreductase, some of these compounds are reduced to aristolochic lactam, while others interact with DNA during the reduction process to form adducts. Nitro groups are critically toxic in AA derivatives, with methoxy and hydroxyl groups further enhancing toxicity. AAI is notably the most toxic element in Aristolochia plants. Further studies have confirmed the strong nephrotoxicity of AA and its metabolite, aristolochic lactam. The mutation and carcinogenic toxicity of AA are linked to its metabolite, AA lactam nitrogen ion, due to its strong electrophilic nature. It can bind to the extracyclic amino groups of DNA bases, forming adducts that may mutate the *RAS* and *p53* genes, subsequently inducing tumors [7,8]. In fact, researchers found the tumor inhibitory activity of AA in the early stage of the study but eventually slowed down due to the toxicity of AA [9,10,11]. In this paper, we summarize the research on the antitumor activity and toxicity of phenanthrenes. To further explore their potential of toxicity-effect transformation, future strategies may include structural modification, traditional Chinese medicine processing techniques or other biological approaches to achieve. In addition, we can gain a more comprehensive understanding through more advanced research design and methods.

## 2. Antitumor Activities of Phenanthrene Alkaloids from Natural Products

**Figure 2:**
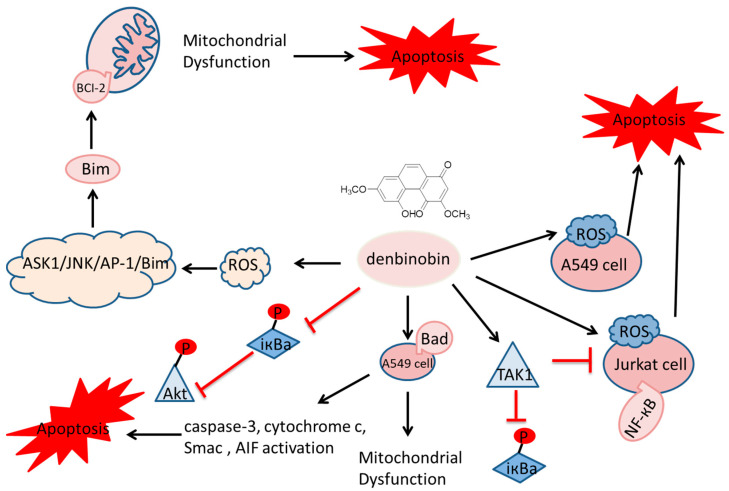
Cytotoxic properties against specific tumor cell lines. 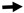 indicate the promotional effects, 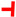 indicate the inhibitory effects.

Phenanthrene and dihydrophenanthrene, isolated from various plants, have demonstrated cytotoxic properties against specific tumor cell lines [12]. In vitro effects may be mediated by several potential mechanisms: cell membrane rupture, impaired cell metabolism, DNA damage or a combination of these. Inducing apoptosis in tumor cells is a primary anticancer strategy and a common mechanism of various anticancer drugs, involving multiple apoptotic mediators that lead to programmed cell death (Figure 2).

### 2.1. Antitumor Phenanthrenes

**Figure 3:**
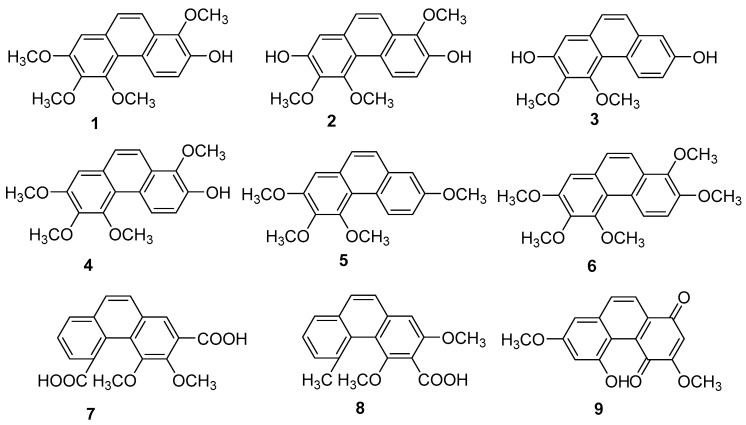
The phenanthrenes in *Dendrobium officinale*.

Orchids are the principal family and the most abundant source of natural phenanthrenes. Twenty-four phenanthrenes have been isolated from Dendrobium genus [12], and there are 12 phenanthrenes in *Dendrobium officinale Kimura & Migo* alone: chrysotoxene (**1**), confusarin (**2**), nudol (**3**), 2,5-Dihydroxy-3,4-dimethoxyphenylene (**4**), 2,3,4,7-tetramethoxy phenylene (**5**), 2,7-Diarboxy-1,5,6-trimethoxyphenanthrene (**6**), 2,5-Dicarboxy-3,4–dimethoxy phenyl (**7**), 3,5-Dicarboxy-2,4-dimethoxyphenyl (**8**) and denbinobin (**9**) (Figure 3). Recent pharmacological studies have revealed their diverse physiological activities, including antitumor, antioxidant and anti-inflammatory effects. Compounds **1**-**3** have exhibited inhibitory effects on liver [13,14], lung [15] and osteosarcoma [16] cancers in vitro. The cytotoxic activity of compound **1** against HepG2 cells has an IC_50_ of 19.64 µM. The IC_50_ values for compound **2** on HI-60 and THP-1 cells are 18.95 ± 0.70 and 11.51 ± 0.12 µM, respectively. For compound **3**, IC_50_ values against the MG63 cell line were 21.86 ± 0.17 µM (24 h), 14.58 ± 0.24 µM (48 h) and 12.97 ± 0.28 µM (72 h); for the U2OS cell line, the values were 21.52 ± 0.08 µM (24 h), 13.99 ± 0.16 µM (48 h) and 11.29 ± 0.21 µM (72 h). Compound **9** has been shown to inhibit NF-κB signaling, exerting anti-inflammatory effects [17,18]. Additionally, compound **2** has been found to promote the growth of neural synapses [19].

**Figure 4:**
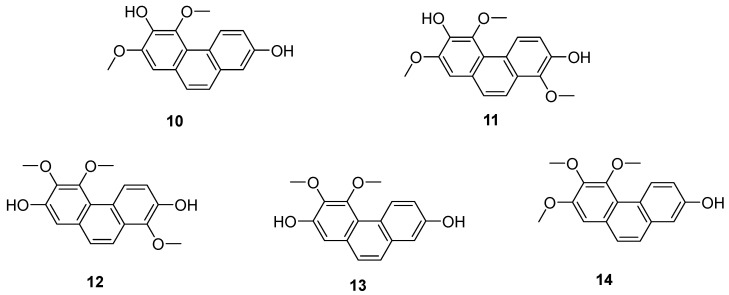
The phenanthrenes in *Tamus communis*.

*Tamus communis*, a member of the Dioscoreaceae family, is native to Asia, Africa and Europe. Five phenanthrenes (**10**–**14**) (Figure 4) were isolated from the fresh rhizomes of Tamus communis by Réthy. They are 7-hydroxy-2,3,4-trimethoxy-phenanthrene (**10**), 2,7-dihydroxy-3,4-dimethoxy-phenanthrene (**11**), 7-dihydroxy-3,4,8-trimethoxyphenan threne (**12**), 3,7-dihydroxy-2,4,8-trimethoxyphenanthrene (**13**) and 3,7-dihydroxy-2,4-dimethoxy phenanthrene (**14**). Compound **10** is a newly discovered natural product, and compounds **11**–**14** were isolated from Tamus communis for the first time. An in vitro MTT assay assessing the cytotoxicity against the HeLa cell line revealed that compound **12** has significant activity, with an IC_50_ value of 0.97 μM [20].

**Figure 5:**
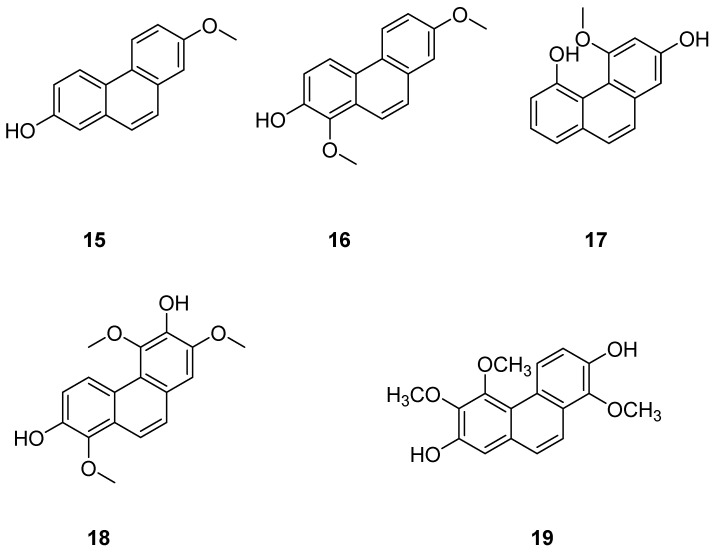
The phenanthrenes in *Dendrobium nobile*.

*Jinchai Dendrobium Lindl.* (*Dendrobium nobile Lindl.*), a valuable medicinal herb within the Orchidaceae family, is part of the Dendrobium genus. Recent research has highlighted phenanthrenes in Dendrobium officinale Lindl. as critical compounds for investigating its anticancer properties, with several exhibiting varying degrees of antitumor activity. Zhou et al. extracted, isolated and purified these natural products from Dendrobium officinale extracts. Subsequently, four phenanthrenes were separated using diverse chromatographic techniques: dentiflor B (**15**), cypripedin (**16**), moscatin (**17**) and 2,4,8-trimethoxyphenanthene-3,7-diol (**18**) (Figure 5). These compounds were tested on human breast cancer MCF-7 cells, demonstrating significant inhibitory effects, which provide robust support for the antitumor research of phenanthrenes in *Dendrobium nobile* [21]. The IC_50_ values for compound **12** on MCF-7, A549 and SW480 cells were 23.75 ± 0.82, 16.29 ± 0.25 and 18.97 ± 1.04 μM [22]. Liang et al. [23] isolated 1,5,6-trimethoxy-2,7-dihydroxy-phenanthrene (**19**) (Figure 5) from Dendrobium officinale, which displayed significant cytotoxic effects on HeLa and HepG2 cells, with IC_50_ values of 0.42 and 0.20 µM.

**Figure 6:**
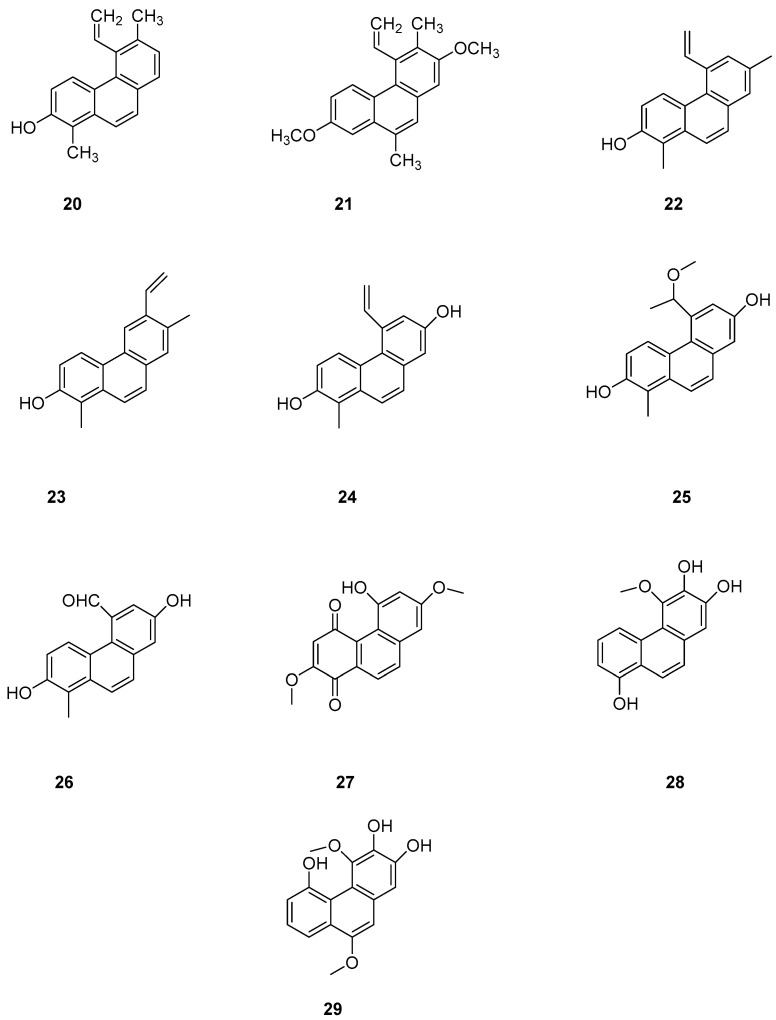
The phenanthrenes in *L. sylvatica*.

Phenanthrenes are prevalent secondary metabolites in plants of the Lamiaceae family. In 2002, Italian scholar Della Greca et al. isolated two new phenanthrenes (**20**–**21**) (Figure 6) and one pyrene compound from *Juncus acutus* L. [24]. In 2020, Ma’s team isolated nine compounds, including coumarins and eight phenanthrenes, from non-polar n-hexane and CH_2_Cl_2_ fractions of *Luzula sylvatica*. Among them, four compounds were newly discovered: hydrojuncinol (**22**) (Figure 6), sylvatin A, sylvatin B and sylvatin C. Phenanthrene has demonstrated promising in vitro anti proliferative activity against various cancer cell lines [25,26,27]. Consequently, the cytotoxicity of these compounds was evaluated for THP-1 (a monocytic leukemia cell line) using a diazazoline assay. The IC_50_ values for compound **22** and hydrojuncuenin (**23**) (Figure 6) were found to be 3 and 5 μM. Moreover, compound **22** significantly inhibited the production of reactive oxygen species (ROS) in a dose-dependent manner, showing moderate cytotoxicity on THP-1 cells with an IC_50_ lower than 6 µM [28]. Further research will focus on the most active compounds, particularly the newly identified phenanthrene dehydrogenated nepenthol, aiming to explore its inhibitory effects on other tumor cells and delve deeper into its mechanism of action. Wen ming Liu et al. isolated and purified 2,7-dihydroxy-1-methyl-5-vinylphenanthrene, named dehydrogenated rush alcohol (**24**) (Figure 6), from traditional Chinese herbal medicine rush, finding that this compound effectively inhibited gastric cancer cell-mediated angiogenesis mimicry with very low toxicity. The activity of SGC-7901 and AGS cells was studied using this compound, and their IC_50_ values were 35.89 µM and 32.92 µM. DHE has demonstrated an inhibitory effect on the growth of gastric cancer cells [29] by inducing tumor-suppressing endoplasmic reticulum (ER) stress responses and reducing tumor-adaptive ER responses [30]. Two compounds, 5-(1-methoxyethyl)-1-methyl-phenanthrene-2,7-diol (**25**) and Dehydroeffusal (**26**) (Figure 6), were isolated from the ethanol extract of *Juncus effuses* by Ma. Cell activity experiments were conducted on SHSY-5Y, SMMC-7721, HepG-2, Hela and MCF-7 cancer cells. These compounds exhibited selective inhibitory activity against MCF-7 cells, with an IC_50_ of 10.9 μM. Compound **26** inhibited the growth of HepG2 and HeLa cells, with very similar IC_50_ values of 12.4 and 13.1 μM, respectively [25]. Denbinobin (**27**), fimbriol B (**28**) and 2,3,5-trihydroxy-4,9-dimethoxy phenanthrene (**29**) (Figure 6) showed a strong ability to induce apoptosis in hepatocytes [31]. Nam et al. studied the cytotoxic effects of nine phenanthrenes and 9,10-dihydro-phenanthrenes on human hypopharyngeal squamous cell carcinoma cell lines, concluding that methylation of the phenolic group or its oxidation to an oxygen-containing group enhances cytotoxic activity [32].

**Figure 7:**
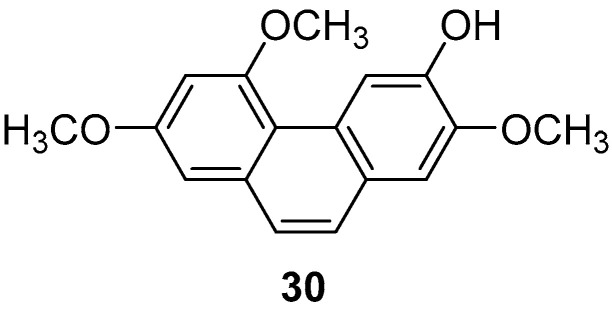
The phenanthrene in yam.

Batatasins, endogenous plant hormones first isolated from the leftover seeds of yam by Hashimoto et al. in 1972 [33], belong to the homodiene class. These molecules feature a variety of hydroxyl and methoxy groups on the A/B ring, with the A ring containing several functional and methoxy groups in the ortho and para-positions. The B ring, however, only contains substitutions in the ortho and meta-positions, lacking oxygen groups [34]. The molecular structure of yam compounds resembles that of resveratrol, which is known for its anti-inflammatory, antioxidant, and anti-tumor properties [35,36]. Consequently, the physiological functions of yam compounds have garnered increasing attention in recent years. Yam Batatasins I (**30**) (Figure 7) was isolated by Min Hye Yang et al. from the chloroform phase of a crude yam extract and found to inhibit both type I and type II cyclooxygenases, with IC_50_ values of 3.0 ± 0.4 and 2.7 ± 0.7 μg/mL [37]. Simultaneously, members of Yue’s research group isolated the same compound from the dichloromethane phase of a crude yam extract, discovering its ability to suppress the production of prostaglandin D2 and leukotriene C4 in mouse bone marrow cells, achieving an IC_50_ value of 1.78 μM [38].

### 2.2. Antitumor 9,10-Dihydrophenanthrene

**Figure 8:**
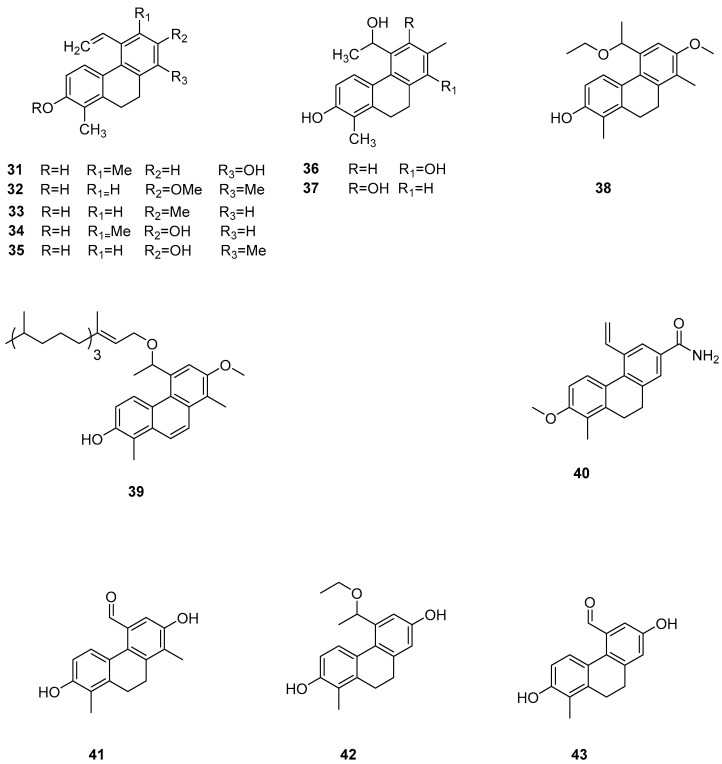
The 9,10-dihydrophenanthrens in *Juncus effuses*.

The search for eco-friendly algae removal agents to manage algal blooms in eutrophic habitats is a prominent area of natural product chemistry. Among these, 9,10-dihydrophenanthrene isolated from the wetland plant *Juncus effusus* is noted for its allelopathic effects on harmful seaweed organisms. In further in-depth research, Marina Della Greca et al. also investigated *Juncus acutus*, which was another wetland cordyceps species from the Mediterranean region, where 9,10-dihydrophenanthrene compounds are significant active ingredients [24]. They reported the separation of nine types of 9,10-dihydrophenanthrene (**31**–**39**) (Figure 8), three phenanthrenes, and one related pyrene compound. Some phenanthrenes isolated from *Juncus effusus* have shown promising cytotoxic and in vitro antitumor activities [39,40]. In 1992, Dellagreca et al. isolated many 9,10-dihydrophenanthrene compounds with in vitro antitumor activity. Csaba Bús et al. assessed these compounds across seven human tumor cell lines (A2780, A2780 cis, KCR, MCF-7, HeLa, HTB-26 and T47D) and one normal cell line (MRC-5) using an MTT assay. The IC_50_ values of compound **22** against these cell lines were 22.3 ± 2.7, 16.9 ± 4.7, 24.2 ± 2.1, 12.9 ± 0.2, 24.7 ± 0.3, 22.8 ± 0.2, 14.2 ± 1.1 and 18.9 ± 4.0 μM, respectively. The IC_50_ values of compound **24** were 23.8 ± 1.3, 37.1 ± 2.8, 35.8 ± 1.7, 37.1 ± 1.1, 0.5 ± 0.0, 41.7 ± 3.5, 25.0 ± 0.4 and 40.9 μM, respectively [41]. Over recent years, several phenanthrenes from the Juncus genus have been tested for their in vitro cytotoxicity against various cancer cell lines using different test systems, exhibiting promising activities. For instance, Su et al. conducted a chemical study on the ethyl acetate-soluble fraction of the ethanol extract from the medulla of rush grass, isolating three new compounds, 9,10-dihydro phenanthrene and rush grass extracts E–G (**40**–**42**) (Figure 8); two new phenanthrene types, dehydrogenated rush grass extracts D–E; a new ferulic glycoside; and a known 9,10-dihydrophenanthrene: 4,7-dihydroxy-2-methoxy-9,10-dihydrophenanthrene (**43**) (Figure 8). They evaluated the in vitro cytotoxic activity of compounds **40**–**43** on seven human cancer cell lines (A549, MCF-7, BEL-7402, HeLa, COLO 205, BGC-823 and SK-OV-3). Among them, compound **40** exhibited weak cytotoxicity on MCF-7 and HeLa cell lines with IC_50_ values of 21.3 and 60.5 μM, respectively. Compound **43** showed moderate cytotoxicity to MCF-7 and HeLa cell lines, with IC_50_ values of 9.17 and 19.6 μM [42]. Lee et al. studied the cytotoxicity of compound **43** and denbinobin, finding that the synthesized methylated and acetylated derivatives did not exhibit antitumor activity in vitro and in vivo [43].

**Figure 9:**
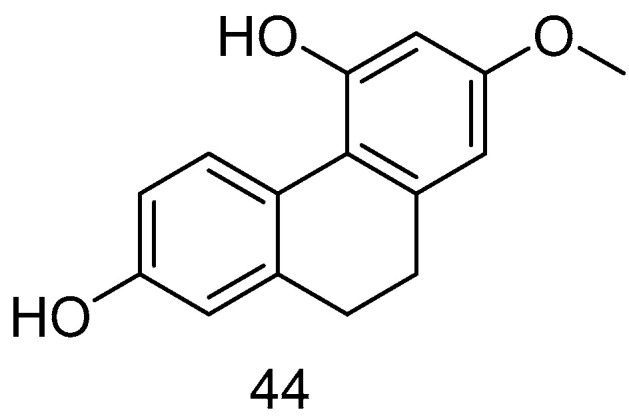
The 9,10-dihydrophenanthrene in *Lusianthridin*.

Lusianthridin (**44**) (Figure 9), isolated from *Dendrobium officinale*, has been demonstrated to exert cytotoxic effects both in vitro and in vivo. This compound shows significant activity against A549 human lung cancer, SK-OV-3 human ovarian adenocarcinoma cells and HL-60 human promyelocytic leukemia cells, with ED_50_ values of 7.7, 9.4 and 9.5 μM [44].

**Figure 10:**
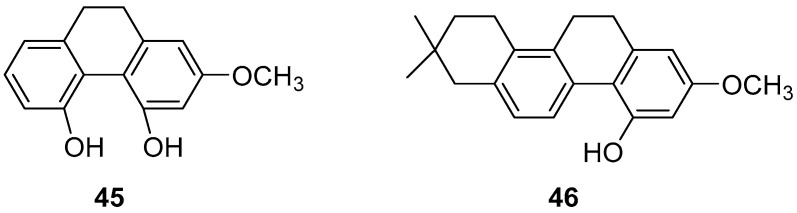
The 9,10-dihydrophenanthrenes in *Dendrobium officinale*.

Zhao et al. conducted studies on various phenanthrene monomers isolated from the rhizomes of *Dendrobium officinale*, revealing significant cytotoxicity against human leukemia cell lines (HI60, THP-1). Orchinol (**45**) (Figure 10) demonstrated substantial cytotoxicity against HI-60 and THP-1 cells, with IC_50_ values of 11.96 and 8.92 μM [13]. Liu et al. isolated 13 phenanthrenes from the orchid plant Panlongshen (*Spiranthes sinensis*) and conducted in vitro cytotoxicity studies on mouse melanoma B16-F10 cells, human gastric cancer SGC-790 cells and human liver cancer HepG2 cells [45]. Their results indicated that spiranthesphenanthrine A (**46**) (Figure 10) exhibited greater cytotoxicity than the control compound cisplatin on B16-F10 cells, with an IC_50_ value of 19.0 ± 7.3 μM. Western blots showed that spiranthesphenanthrine A inhibits the migration of B16-F10 cells, potentially due to the inhibition of epithelial cell apoptosis. This compound may be a potential candidate for preventing tumor metastasis.

**Figure 11:**
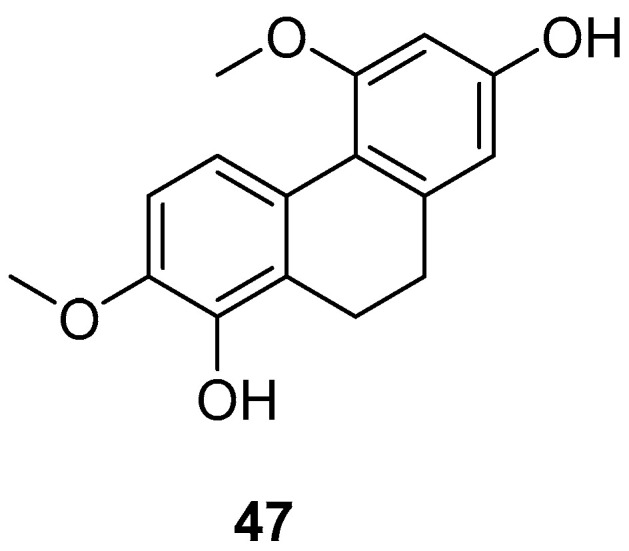
The 9,10-dihydrophenanthrene in *Bai Ji*.

*Bai Ji* (*Bletilla striata*), a traditional Chinese medicine, comprises the dried tubers of the orchid plant *Bletilla striata (Thunb.) Reichb. f*., a prized medicinal variety mainly found in East China, Central South, Southwest, Gansu, Shaanxi and other regions. Guizhou is noted for the largest production and best quality. Annually from September to October, as the stems and leaves wither, the tubers are harvested, roots removed, washed, boiled or steamed until no white core remains, and then sun-dried until semi-dry, after which the outer skin is removed, and further sun-drying is conducted. As of December 2020, over 261 compounds have been isolated and identified from this genus. These isolates include stilbenes (benzyl and phenanthrene), flavonoids, triterpenoids, steroids, simple phenolic compounds and glucosoxybenzyl 2-isobutyl malate compounds. The 9,10-dihydro-4,7-dimethoxyphenanthrene-2,8-diol (**47**) (Figure 11) has shown inhibitory effects on LPS-induced NO production in RAW 264.7 cells, with an IC_50_ value ranging from 25.0 to 87.2 μM [46].

**Figure 12:**
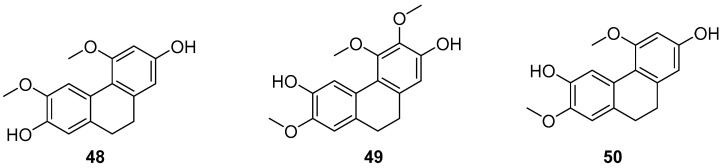
The 9,10-dihydrophenanthrenes in *Combretum laxum*.

Combretum, a genus in the Loricaceae family, comprises approximately 250 species distributed across both tropical hemispheres, excluding Oceania, with a significant presence in tropical Africa. About 12 species are found in China, primarily south of the Yangtze River. The family Serranidae, to which it belongs, is known for yielding a variety of bioactive chemical components. Eder Bisoli et al. conducted chemical analyses on the roots and stems of *Combretum laxum*, isolating a new dihydrostilbene derivative, two phenanthrenes, three dihydrophenanthrenes (**48**–**50**) (Figure 12), along with a lignan, a triterpene, an orange ketone, a flavone, a naphthoquinone and two benzoic acid derivatives [47]. The appearance of these compounds in the genus Windmill is unprecedented in the Americas. Compounds 2,7-dihydroxy-4,6-dimethoxyphenylene (**48**) and 2,6-dihydroxy-4,7-dimethoxy-9,10-dihydrophenanthrene (**50**) are novel discoveries in the Combretaceae family. These compounds were tested for in vitro cytotoxicity against five human cancer cell lines and for their free radical scavenging ability against 1,1-diphenyl-2-picrylhydrazine (DPPH). Compound **48** exhibited the most potent cytotoxicity against melanoma cells (IC_50_ of 2.59 ± 0.11 µM) and showed high selectivity compared to its effects on non-tumor mammalian cells (SI25.1).

**Figure 13:**
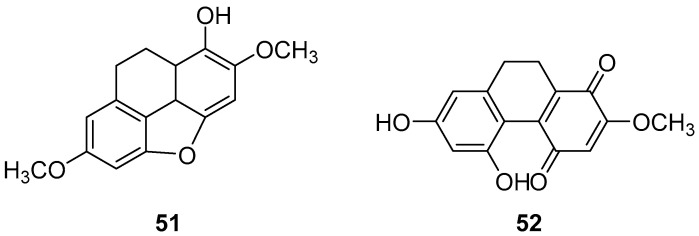
The 9,10-dihydrophenanthrenes in *Pholidota chinensis*.

*Pholidota cantonensis Rolfe*, a perennial herbaceous plant in the Orchidaceae family with over 30 species, is native to regions in China such as Zhejiang, Jiangxi, Fujian, Taiwan, Hunan, Guangdong and Guangxi. Li et al. isolated two 9,10-dihydro phenanthrene compounds, phytol (**51**) and phocantone (**52**) (Figure 13), from the ethanol extract of the whole plant in Guangzhou. These compounds were evaluated for their cytotoxic activity against mouse leukemia P388D1 cancer cells, with IC_50_ values of 75.0 and 27.5 µM, respectively [48].

**Figure 14:**
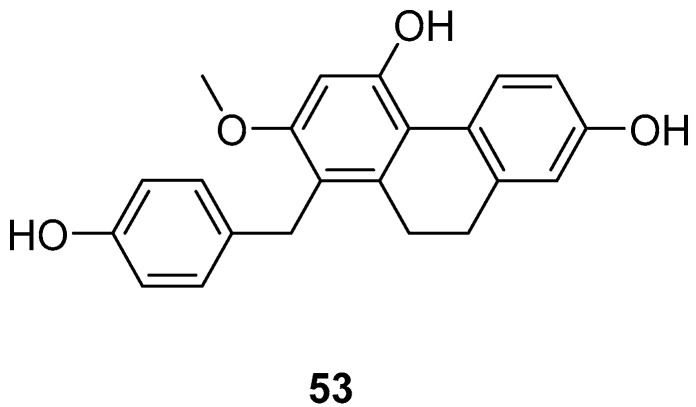
The 9,10-dihydrophenanthrene in *Cymbidium hybridum*.

*Cymbidium hybridum*, an evergreen epiphytic herb from the Orchidaceae family, is predominantly found in East Asia, including China, Japan and South Korea, where it has been cultivated for centuries [49]. Shuang-shuang Lv extracted compounds from *C. grandiflora* and assessed their effects on human cancer cell lines using the MTT method. Shancidin (**53**) (Figure 14) was found to significantly impact all three cancer cell lines tested. The IC_50_ values for SMMC-7721, A549 and MGC80-3 cells were 12.57, 18.21 and 11.6 μM, respectively [50].

### 2.3. Antitumor 9,10-Dihydrophenanthrene Dimer Compounds

**Figure 15:**
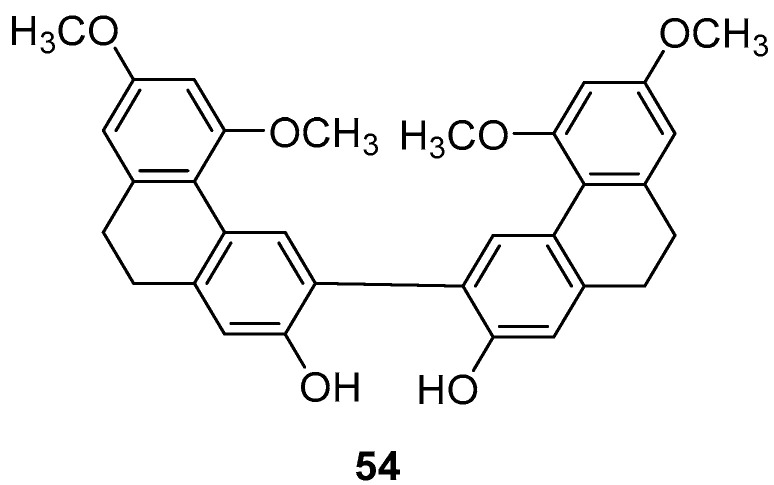
The 9,10-dihydrophenanthrene dimer compound in *Panlong ginseng*.

Panlong ginseng, a traditional Chinese medicine name, refers to the root or entire plant of *Spiranthes australis Lindl.* from the Orchidaceae family, distributed across various Chinese provinces and regions. Liu et al. extracted a 9,10-dihydrophenanthrene dimer compound (**54**) (Figure 15) and studied its activity, finding that the IC_50_ values for SGC-7901, HepG2 and B16-F10 cells were 63.8 ± 3.6, 78.4 ± 29.0 and 58.2 ± 2.6 µM, respectively [45].

**Figure 16:**
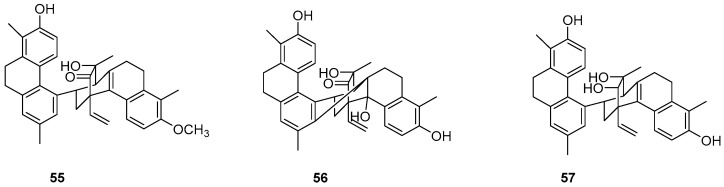
The 9,10-dihydrophenanthrene dimer compounds.

The potential of natural compounds as anticancer agents has attracted researchers like Chiara Platella et al. to expand the library of natural compounds, particularly phenanthrenes, using biophysical and molecular docking techniques. They synthesized a dimer containing 9,10-dihydrophenanthrene (**55**–**57**) (Figure 16) and assessed its antiproliferative activity on HeLa adenocarcinoma, MCF7 breast cancer and A431 epidermoid carcinoma cells via the MTT method. The IC_50_ values for compound **56** on HeLa, MCF-7 and A431 cells were 25, 31 and 42 μM, respectively [51].

**Figure 17:**
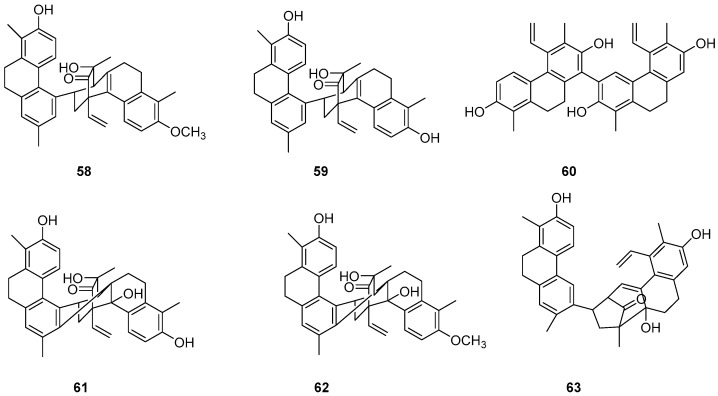
The 9,10-dihydrophenanthrene dimer compounds in the *Dendrobium genus*.

**Figure 18:**
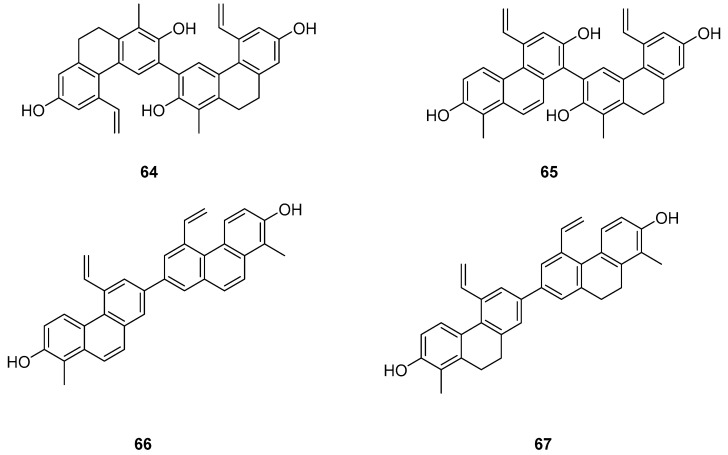
The 9,10-dihydrophenanthrene dimer compounds in *Juncus effuses*.

**Figure 19:**
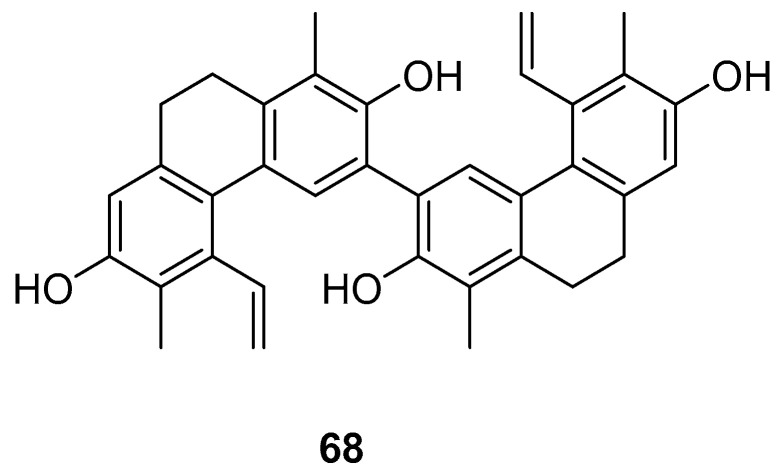
The 9,10-dihydrophenanthrene dimer compound in *J. compressus*.

The Dendrobium genus comprises perennial, primarily herbaceous plants with around 240 species distributed globally, predominantly in temperate and cold regions. These plants typically thrive along the water’s edge in grassy swamps and damp environments. In recent years, new structures of dimeric 9,10-dihydrophenanthrenes have been isolated from the Juncus plant. From 1997 to 2002, Dellagreca et al. identified only five types of dimeric phenanthrenes in *Juncus acutus* [52]. In 2003, six new dimeric 9,10-dihydrophenanthrene compounds (**58**–**63**) (Figure 17) were isolated from this species [53]. Diphenanthrenes are rare in this genus, with nine reported from *Jasminum acuminatum* and one from *Wild jasmine* [52,54,55]. In the process of bioactive natural products from traditional Chinese medicine, Wei Ma isolated four new phenanthreneoid dimers from the ethanol extract of *Juncus effuses*’ medulla in 2015, named effususins A–D (**64**–**67**) (Figure 18). These compounds’ effects on cell proliferation and survival were measured in SHSY-5Y, SMMC-7721, HepG-2 Hela and MCF-7 cells using the CCK-8 method, with paclitaxel as the positive control. Among the phenanthrene dimers, compound **64** showed moderate to strong cytotoxic activity against all five cancer cell lines, with IC_50_ values of 32.64, 13.60, 12.93, 25.09 and 12.49 µM, respectively, outperforming paclitaxel except against the SMMC-7721 cell line. The other three compounds exhibited no activity against these cell lines [56]. Csaba Bús et al. extracted a new dimeric phenanthrene, named compressin B (**68**) (Figure 19), from *Juncus compressus Jacq.* and tested its antiproliferative activity against three human tumor cell lines: HeLa and SiHa (cervical adenocarcinoma) and A2780 (ovarian cancer). The HeLa cell line was the most sensitive, with an IC_50_ value of 1.86 μM [57].

### 2.4. Other Derivatives of Antitumor Phenanthrenes

**Figure 20:**
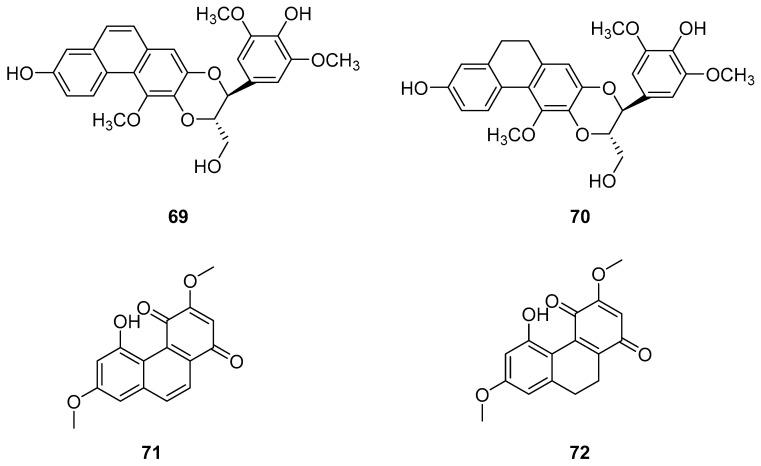
The other derivatives of phenanthrenes in *Dendrobium*.

Dendrobium, one of the largest genera in the Dendrobium family, consists of over 1100 species, most of which are found in Asia, Europe and Australia [58]. In China, there are 74 species and two varieties, several of which are utilized in traditional or folk medicine. Phenanthrenes [59] and sesquiterpenes isolated from these plants exhibit significant antioxidant, anti-tumor, immunomodulatory and anti-inflammatory activities [60,61,62,63]. Zhao et al. isolated two new phenanthrenes and 9,10-dihydrophenanthrene derivatives (**69**–**70**) (Figure 20), along with six known homologues, from *Dendrobium officinale* stem extracts. Cell studies conducted on HI-60 and THP-1 cell lines revealed IC_50_ values of 35.32 ± 1.76, 20.78 ± 1.80, >50 and 45.32 ± 2.39 µM for compounds **69** and **70**, respectively [13]. Denbinobin (**71**) (Figure 20), a phenanthrenequinone from the Dendrobium genus, can be extracted from *D. candidum* [64,65], *D. nobile* [18,59,66,67] and *D. venustum* [68]. Ephemeranthoquinone (**72**) (Figure 20) is also derived from *D. hancockii*, *D. hongdie* [69], *D. longicornu* [70,71], *D. plicatile* [72] and other plants. Zhai summarized the IC_50_ values of these compounds on MCF-7, HL-60, A549 and SW480 cells as 13.13 ± 0.47 and 3.63 ± 0.03, 3.08 ± 0.12 and 2.33 ± 0.12, 19.68 ± 1.12 and 14.79 ± 0.64 and 16.81 ± 0.13 and 6.66 ± 0.71 μM, respectively [73].

**Figure 21:**
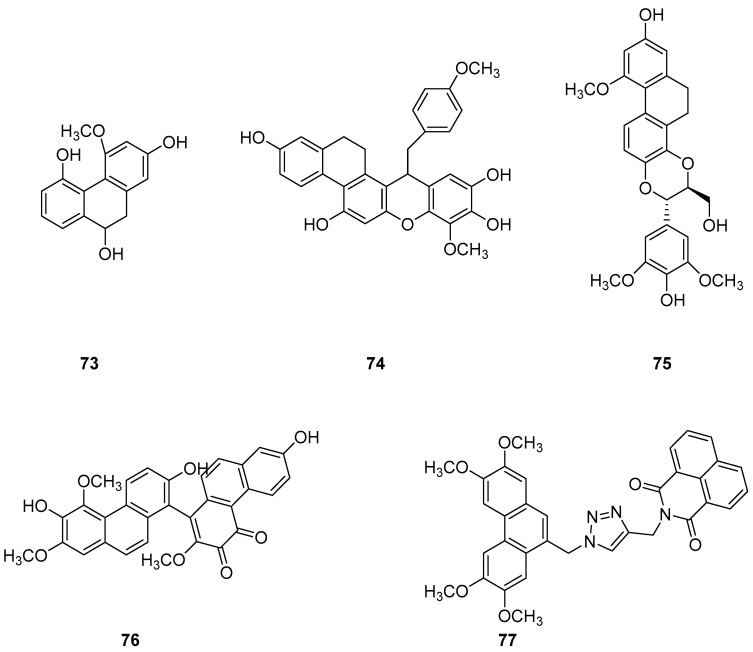
The other derivatives of phenanthrenes in *Indian orchids*.

**Figure 22:**
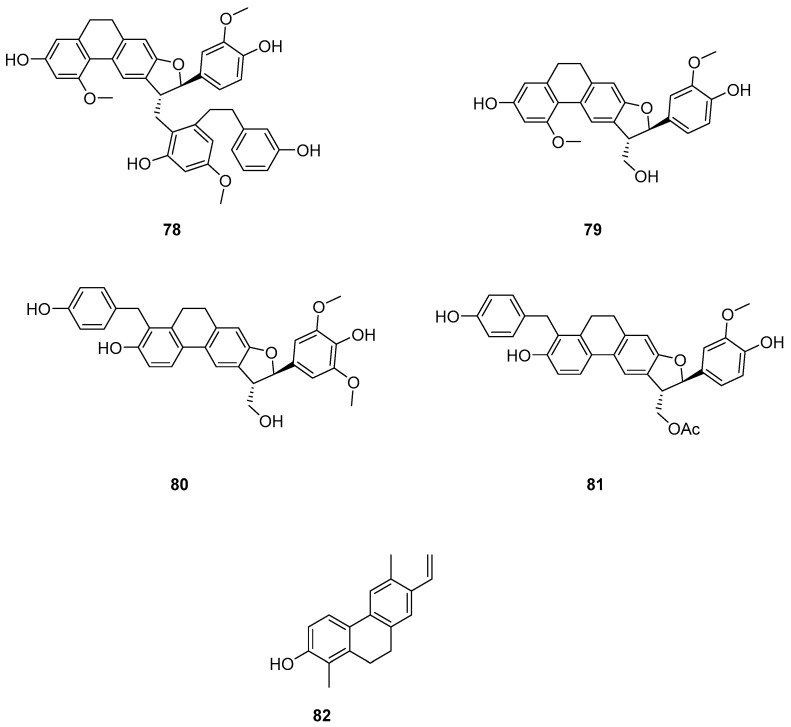
The other derivatives of phenanthrenes in *Bletilla striata*.

**Figure 23:**
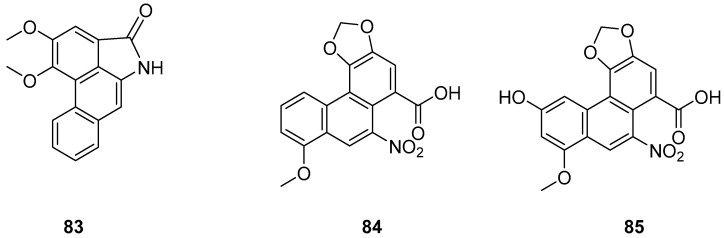
The other derivatives of phenanthrenes in *Aristolochia*.

The earlier literature reports the isolation of significant quantities of stilbenes, steroids and triterpenes from a series of Indian orchids (*Dendrobium rotundatum*), which also contain a large amount of biphenyl, phenanthrene, phenanthropyran and pyran compounds, including 9,10-dihydrophenanthrene derivatives. Majumder continued to explore these phytochemicals and isolated a new 9,10-dihydrophenanthrene derivative from Dendrobium officinale, named rotundatin (**73**) (Figure 21) [74]. Over the past five years, numerous new phenanthrenes derivatives have been isolated from natural plants and their anticancer activities tested [75]. In 2016, a new biphenylphenanthrene compound **74** named 8-methoxy-12-(4-methoxybenzyl)-13,14-dihydro-12*H*-naphtho[2,1-a] xanthene-2,5,9,10-tetraol was isolated from *Dendrobium officinale*, displaying appropriate antiproliferative activity against MDA-231, HepG2 and HT-29 cancer cells, with IC_50_ values of 25.2, 51.3 and 30.4 μM [76]. Compound **75**, a novel phenanthrene derivative isolated from *Eria bambusifolia* (*Callostylis bambusifolia* (*Lindl.*)) in 2016, showed effective antiproliferative activity against HL-60 cancer cells, with an IC_50_ of 14.5 μM [77]. That same year, a new phenanthrene derivative (**76**) (Figure 21) was isolated from *Bai Ji*, demonstrating potent inhibitory effects on MCF-7, HT-29, HUVEC and A549 tumor cells, with IC_50_ values of 12.6, 22.7, 33.5 and 22.6 μg/mL [78]. Further research indicated that compound **76** induces the stagnation of cancer cell division in the G0/G1 phase, inhibiting the transition from G1 to S phase. In 2017, six phenanthrenes were isolated from the stems of *Dendrobium officinale*, showing significant antiproliferative activity against HL-60 and THP-1 cancer cells [13]. Among them, compound **77** predominantly inhibits the growth of DU145 cells by arresting them in the G0/G1 phase of mitosis, with an IC_50_ of 1.5 μM [79]. In 2018, Li et al. extracted four new dihydrophenanthrofuran compounds, named bleochranols A–D (**78**–**81**) (Figure 22), from the roots of *Bletilla striata*. Of these, compound **80** exhibited the most potent activity. The IC_50_ values of bleochranol A against HL-60, SMMC-7721, A-549, MCF-7 and SW480 cells were 0.24 ± 0.03, 12.22 ± 0.26, 3.51 ± 0.09, 3.30 ± 0.99 and 12.97 ± 0.34 μM, respectively [80]. Juncunol (**82**) (Figure 22) was isolated from Juncus effusus L. and found to induce apoptosis in the human hepatoma cell line HepG2 [81]. Zhang et al. conducted cytotoxicity tests, revealing that the compounds AL-BII (**83**), AAI (**84**) (Figure 23) and AMH extracts exhibited notable cytotoxicity against HepG2 cells, with IC_50_ values of 0.2, 9.7 and 50.2 μM. AAI showed specific cytotoxicity to HepG2 cells, whereas AAD (**85**) (Figure 23) displayed no cytotoxicity. Among the AA derivatives, AL-BII demonstrated the strongest cytotoxicity and selectivity towards the NCI-H187 cell line but was non-toxic to A549 and MCF7 cells [82].

## 3. Toxicological Effects of Natural Phenanthrenes

Aristolochic acid (AA), a natural nitrophenanthrene carboxylic acid compound, consists of AAI and AAII in a ratio of approximately 1:1 [83]. These compounds are present in numerous plants, including *Aristolochia, Asarum, Akebia, Clematis, Stephania, Menispermum, Dauricum* and *Asteraceae*. AA has been utilized pharmacologically for a long time and was used to treat edema over 2500 years ago in ancient Greek and Egyptian practices [84]. In China, the medicinal plants containing AA are mainly from the Aristolochia and *Asarum* species. Several plants from the *Aristolochiaceae* family are used in traditional medicine globally, such as the fruit of *Aristolochia*, the canes of *Caulis aristolochiae manshuriensis*, the roots of *Stephania tetrandra* and *Radix aucklandiae*. Modern studies have shown that Aristolochia herbs possess diuretic, anti-infective, anti-inflammatory, anti-venom and anticancer properties [85,86,87,88]. It has also been documented that plants or plant-derived products containing AA are toxic, mutagenic and carcinogenic to humans [89,90], leading to a ban on their use in some countries.

### 3.1. Hepatotoxicity

**Figure 24:**
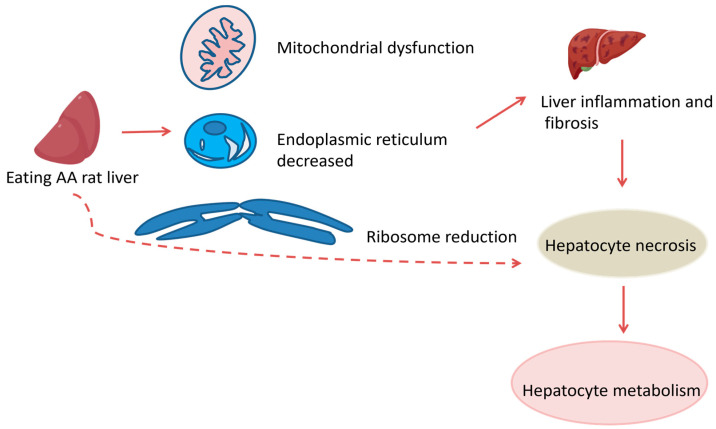
Hepatotoxicity in rats. indicate the inhibitory effects. 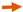 indicate the promotional effects.

AA is a key component in Chinese herbal medicines such as AA and asarum, commonly found in Southern Brazil and India. These herbs are used to treat various ailments including eczema, pneumonia, stroke, hepatitis, snakebite, arthritis, gout and coronary artery disease. Research published on 18 October 2017, by scientists from Singapore and Taiwan indicated that AA was responsible for a majority of hepatocellular carcinoma (HCC) cases in Taiwan [91]. In 2019, Ouyang monitored the metabolism of AAI and its residues in the liver and kidneys of rats fed with AAI, discovering that AAI metabolized in the kidney while AAT residues were found in both the liver and kidney [92]. In 2021, Wang et al. conducted toxicity tests on rats with varying doses of AA, finding that increased doses resulted in liver mitochondrial damage, reduction in organelles such as ER and ribosomes, liver inflammatory cell infiltration and a tendency toward fibrosis. These findings indicate that AA can cause hepatocyte necrosis and subsequently impair hepatocyte metabolic function. The prolonged and high-dose usage of AA leads to severe liver damage in a dose-dependent manner [93]. This aligns with Quan et al.’s 2020 findings that AA-containing herbs damaged mouse kidney mitochondria [94]. AA induces liver injury through oxidative stress and mitochondrial apoptosis pathways. The results showed that AST and ALT levels in serum were significantly elevated in all dose groups compared to the control, confirming dose-dependent toxicity [95]. In Wang’s study, the highest serum AST and ALT levels were observed in the 20 mg/kg-AA dose group, confirming the severe hepatic damage at this concentration [93]. Oxidative stress, primarily occurring in mitochondria, is a major pathological factor in many organisms and can lead to apoptosis [96]. The mitochondrial-mediated apoptosis pathway is one of the classic routes of apoptosis [97], which are critical factors in AA-induced hepatotoxicity (Figure 24).

### 3.2. Nephrotoxicity

In 1994, Cosyns et al. began to suspect that AA could cause kidney disease [98]. Debelle et al. linked the consumption of AA-containing herbs to Chinese herbal nephropathy (CHN)/AA nephropathy (AAN) in 2007, which is a progressive interstitial nephritis leading to end-stage renal disease and urothelial malignancies [99]. CHN was initially identified in Belgium as a new cause of chronic renal interstitial fibrosis associated with weight loss programs that included Chinese herbal medicine [100]. Subsequently, AA was confirmed as the pathogenic factor [101,102], and CHN was more accurately described as AAN [103,104]. In Southeastern Europe, a similar condition previously identified as Balkan endemic nephropathy (BEN) was later also recognized as AAN [105]. Following the recognition of AAN in the 1990s, numerous studies have elucidated the cellular and molecular mechanisms underlying the condition.

**Figure 25:**
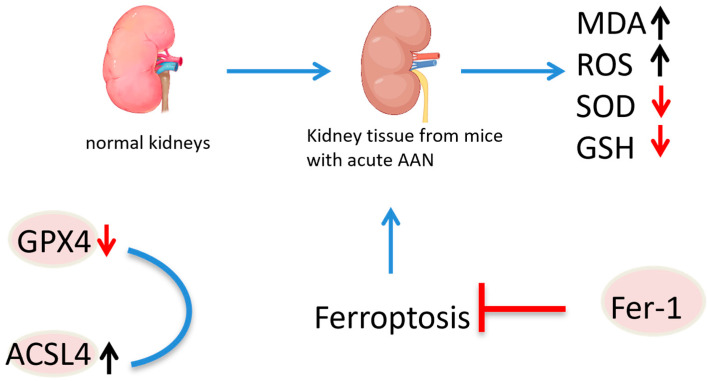
Mechanism of aristolochic acid causing nephrotoxicity. 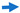 indicate the promotional effects, 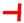 indicate the inhibitory effects, 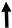 indicate the promotional effects, 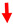 indicate the inhibitory effects in the figure.

Ferroptosis, characterized by iron-dependent lipid peroxidation, requires sufficient free intracellular iron and polyunsaturated fatty acids (PUFAs) in the cell membrane. Acyl-CoA synthetase long-chain family member 4 (ACSL4) and lysophosphatidylcholine acyltransferase 3 (LPCAT3) are involved in the synthesis and remodeling of PUFA-phosphatidylethanolamine (PE) in cell membranes. The inhibition of ACSL4, but not other ACSL family members, prevents ferroptosis. GPX4, a major endogenous antioxidant enzyme, prevents the accumulation of toxic lipid ROS during ferroptosis [106]. The depletion of glutathione (GSH) and inactivation of GPX4 lead to fatal iron-dependent accumulation of lipid ROS. The conditional knockout of GPX4 is linked with cancer, neurodegenerative diseases, acute kidney injury or liver injury, preventable or mitigated by inhibiting ferroptosis [106,107,108,109]. Acute AAN in mice has shown increased malondialdehyde (MDA) content, enhanced lipid peroxidation, decreased superoxide dismutase (SOD) activity, depleted GSH, and impaired antioxidant capacity. The expression of GPX4 was reduced, while that of ACSL4 was increased, suggesting the involvement of ferroptosis in AA-induced acute kidney injury. Ferroptosis inhibition by Fer-1 significantly improved renal function, reduced histopathological lesions, decreased lipid peroxidation and restored antioxidant capacity. In vitro, AA markedly reduced cell viability, induced ROS production, increased intracellular iron levels and decreased iron poisoning-related protein expression. Ferroptosis inhibition notably increased cell viability and mitigated AA-induced renal tubular epithelial cell injury [110]. As early as 2013, it was reported that AA could induce apoptosis in renal tubular epithelial cells (TECs) through the dephosphorylation of signal transducer and activator of transcription 3 (STAT3) and activation of the *p53* signaling pathway (Figure 25) [111].

**Figure 26:**
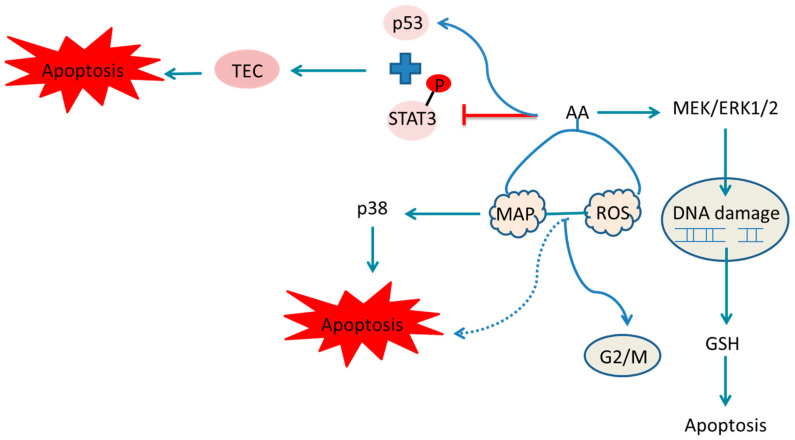
Mechanism of aristolochic acid causing cellular damage and DNA damage. 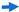 indicate the promotional effects and 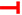 indicate the inhibitory effects in the figure.

ROS or reactive nitrogen species (ROS/RNS) are products of normal cell metabolism, playing a crucial role in cell signal transduction and homeostasis. The excessive ROS/RNS production or depletion of endogenous antioxidant systems can lead to oxidative/nitrosative stress [112]. Exogenous substances like AA can also induce oxidative stress, resulting in cellular damage [113]. AA has been shown to damage DNA by stimulating ROS production. Yu used human cells to further explore the mechanism of AAI-induced DNA oxidative damage [114]. Romanov et al. noted that AA could induce apoptosis and cell arrest in the G2/M phase by generating ROS and activating the mitogen-activated protein kinase (MAP kinase), which in turn activates p38, leading to apoptosis (Figure 26) [48].

Declèves’ experiments demonstrated inflammation-related oxidative stress in the AA-induced acute kidney injury mouse model [115]. Jin et al. found that AAI-induced oxidative stress in C57BL/6N mice was associated with the increased expression of NADPH oxidase 2 (NOX2), CYP2E1 and decreased catalase, SOD and glutathione synthetase levels, suggesting AA may interact with these enzymes to induce oxidative stress [116]. Research has indicated that mitochondria are a target of AA-induced nephropathy. Pozdzick’s data revealed AA toxicity primarily causes mitochondrial damage and impairs the activation of antioxidant enzymes. Additionally, Zhou et al. found that AA promoted mitochondrial DNA (mtDNA) damage, reduced the mtDNA copy number and decreased mitochondrial protein expression in cultured podocytes. AA treatment also lowered ATP content, the oxygen consumption rate and mitochondrial membrane potential in these cells and increased cellular ROS [117]. Furthermore, AA has been shown to induce apoptosis in renal tubular cells by inhibiting the PI3K/Akt signaling pathway [118,119], decreasing Bcl-2 levels and increasing Bcl-2 associated X protein (Bax) levels in human umbilical vein endothelial cells (HUVECs) [120]. Zhu and Hsin linked ER stress with AAI-induced apoptosis [121,122]. AAN is also associated with inflammation, as evidenced by the presence of mononuclear inflammatory cells in chronic renal scars in rats [123]. Honarpisheh’s data suggest that macrophages play a crucial role in AAN’s pathogenesis, related to excessive macrophage accumulation and activation [124]. AA-treated zebrafish embryos displayed inflammation, indicated by upregulated *pro-inflammatory gene* expression [125]. Similarly, Jadot observed increased renal mRNA expressions of pro-inflammatory cytokines in AA-treated mice [126]. Recent studies have also linked the NLRP3 inflammasome with AAN [127]. Kholia et al. and Wang et al. reported inflammation in AA rat models. These findings collectively demonstrate that AA can cause kidney disease, renal failure, and upper urinary tract cancer [128,129].

### 3.3. Carcinogenicity

**Figure 27:**
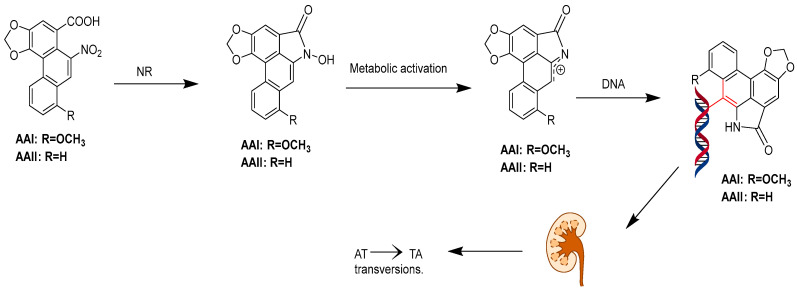
Pathways for the metabolic activation of the aristolochic acids. The highlighted parts indicate the binding of aristolochic acid metabolites to DNA.

Studies from the 1990s and early 21st century identified AA as a potent carcinogen and renal toxin, leading to its classification as a human carcinogen by the International Agency for Research on Cancer (IARC) in 2012. Upper urinary tract urothelial carcinoma (UC) is commonly found in patients with end-stage AA-related nephropathy, often occurring long after the initial onset of nephropathy. Both upper urinary tract and bladder UC are severe complications in renal transplant recipients with acid nephropathy, confirming the carcinogenic properties of AA [130]. Kwok’s research suggests that UC in traditional Chinese medicine is associated with AA exposure, potentially through skin contact or the inhalation of fine powder [131]. A recent comprehensive epidemiological study demonstrated that the use of herbal products containing AA is significantly linked with a higher dose-dependent risk of various cancers including liver, colorectal, kidney, bladder, prostate, pelvic and ureteral cancer. An increased risk of extrahepatic cholangiocarcinoma was observed particularly in women exposed to higher doses of AA, highlighting its genotoxicity [132]. AA is metabolized to form AA lactam (AL), which binds to DNA, preferentially to adenine and guanine. This binding induces errors in DNA synthesis, leading to substitutions of G:C to T:A and A:T to T:A. However, the AL–guanine adduct is recognized by global genomic nucleotide excision repair (GG-NER), making T > A substitutions a characteristic mutation of AA, typically found at the 5’-pyrimidine-A-purine-3’ site. This ultimately causes DNA damage and triggers liver cancer [133,134,135]. Additionally, AL-DNA adducts formed in the kidneys can cause renal tubular injury and renal interstitial fibrosis, persisting long-term, exacerbating renal damage and significantly increasing the risk of kidney disease and upper urinary tract epithelial cancer (Figure 27).

## 4. Conclusions and Prospect

With advancements in science and technology increasing health awareness, natural plant extracts have gained significant attention. As vital sources of natural medicines, these extracts have broad applications. In this paper, we summarized 84 natural phenanthrenes and their derivatives (Table 1). Phenanthrenes, primary active compounds found in plants like Dendrobium and Bletilla, have drawn considerable interest from scientists due to their anti-tumor potential and toxic effects.

Historically, research on phenanthrenes has primarily focused on separation, extraction, pharmacological activities, mechanisms of action and chemical synthesis [136]. Currently, in China, natural plants containing phenanthrenes are well recognized medically among the public, yet knowledge of their chemical composition and active pharmacological substances remains limited. Phenanthrenes are significant chemical components of families like Caryophyllaceae and Orchidaceae, with more than 450 species compounds that have been isolated since the 1970s. It is important to note that almost all of the phenanthrene compounds have been studied for their potential biological activity, and several have shown multiple activities. During the screening process, some serve as promising resources for innovative drug lead compounds. Denbinobin, for instance, shows potential as a lead anticancer compound with a novel mechanism of action. AA, a nitrophenic acid compound, can cause various cancers such as urothelial, renal cell, hepatocellular, biliary tract and gastrointestinal cancers when ingested through Chinese herbal medicines, skin contact, or the inhalation of herbal powder [4]. In the future, we can transform its toxic effects into anticancer properties by modifying its structure. Therefore, we need new technologies to identify novel targets, such as PROTAC probe technology [137].

Although aristolochic acid compounds are highly toxic, we can engineer them through multiple pathways that may contribute to their ultimate pharmacological use in drug therapy. After that, we will work with other disciplines to study its mechanism of toxic effect conversion in multiple directions. In addition, we can perform structural modification, traditional Chinese medicine processing techniques or other biological approaches to achieve the toxicity-effect transformation of Aristolochic acid. The development of these new treatment strategies can achieve the effect of toxic effect conversion and provide new ideas for the treatment and development of phenanthrenes, so as to be better used in clinical practice in the future.

## Figures and Tables

**Table 1:** Anticancer activity of phenanthrene natural products and their derivatives.

Compounds	Anticancer Activity	Source	References
Chrysotoxene (**1**)	IC_50_ (HepG2) = 19.64 µM.	*Dendrobium genus*	[12,13,14,15,16]
Confusarin (**2**)	IC_50_ (HI-60) = 18.95 ± 0.70 µM; IC_50_ (THP-1) = 11.51 ± 0.12 µM.	*Dendrobium genus*	[12,13,14,15,16]
Nudol (**3**)	IC_50_ (MG63) = 12.97 ± 0.28 µM; IC_50_ (U2OS) = 11.29 ± 0.21 µM.	*Dendrobium genus*	[12,13,14,15,16]
7-dihydroxy-3,4,8-trimethoxyphenan threne (**12**)	IC_50_ (HeLa) = 0.97 µM.	*Tamus communis*	[20]
Moscatin (**17**)	IC_50_ (MCF-7) = 23.75 ± 0.28 µM; IC_50_ (A549) = 16.29 ±0.25 µM; IC_50_ (SW480) = 18.97 ± 1.04 µM.	*Dendrobium nobile*	[21,22]
1,5,6-trimethoxy-2,7-dihydroxy- phenanthrene (**19**)	IC_50_ (HeLa) = 0.42 µM; IC_50_ (HepG2) = 0.2 µM.	*Dendrobium officinale*	[23]
Hydrojuncinol (**22**)	IC_50_ (THP-1) = 3 µM.	*L. sylvatica*	[25,26,27,28]
Hydrojuncuenin (**23**)	IC_50_ (THP-1) = 5 µM.	*L. sylvatica*	[25,26,27,28]
Dehydrogenated rush alcohol (**24**)	IC_50_ (SGC-7901) = 35.89 µM; IC_50_ (AGS) = 32.92 µM.	*Traditional Chinese herbal medicine rush*	[29]
5-(1-methoxyethyl)-1-methyl-phenanthrene-2,7-diol (**25**)	IC_50_ (MCF-7) = 10.87 ± 0.82 µM; IC_50_ (HepG2) = 37.03 ± 2.44 µM; IC_50_ (HeLa) = 52.82 ± 5.58 µM; IC_50_ (SHSY-5Y) = 31.98 ± 2.64 µM; IC_50_ (SMMC-7721) = 42.94 ± 2.95 µM.	*J. effuses*	[30]
Dehydroeffusal (**26**)	IC_50_ (MCF-7) = 45.83 ± 5.54 µM; IC_50_ (HepG2) = 12.43 ± 2.56 µM; IC_50_ (HeLa) = 13.07 ± 2.56 µM; IC_50_ (SHSY-5Y) = 30.05 ± 1.64 µM; IC_50_ (SMMC-7721) = 25.35 ± 2.08 µM.	*J. effuses*	[30]
Extract I (**30**)	IC_50_ (prostaglandin D2 and leukotriene C4) = 1.78 µM.	*Batatasins*	[33,37,38]
7-methoxy-1,8-dimethyl-5-vinyl-9,10-dihydrophenanthren-2-ol (**32**)	IC_50_ (A2780) = 22.3 ± 2.7 µM; IC_50_ (A2780 Cis) = 16.9 ± 4.7 µM; IC_50_ (KCR) = 24.2 ± 2.1 µM; IC_50_ (MCF-7) = 12.9 ± 0.7 µM; IC_50_ (HeLa) = 24.7 ± 0.3 µM; IC_50_ (HTB-26) = 22.8 ± 0.2 µM; IC_50_ (T47D) = 14.2 ± 1.1 µM; IC_50_ (MRC-5) = 18.9 ± 4.0 µM.	*Juncus acutus*	[24,41]
1,6-dimethyl-5-vinyl-9,10-dihydrophenanthrene-2,7-diol (**34**)	IC_50_ (A2780) = 23.8 ± 1.3 µM; IC_50_ (A2780 Cis) = 37.1 ± 2.8 µM; IC_50_ (KCR) = 35.8 ± 1.7 µM; IC_50_ (MCF-7) = 37.1 ± 1.1 µM; IC_50_ (HeLa) = 0.5 ± 0.0 µM; IC_50_ (HTB-26) = 41.7 ± 3.5 µM; IC_50_ (T47D) = 25.0 ± 0.4 µM; IC_50_ (MRC-5) = 40.9 µM.	*Juncus acutus*	[24,41]
Juncuenins E (**40**)	IC_50_ (MCF-7) = 21.3 µM; IC_50_ (HeLa) = 60.5 µM.	*Juncus effuses* L.	[42]
Juncuenins F (**41**)	IC_50_ (MCF-7) > 100 µM; IC_50_ (HeLa) > 100 µM.	*Juncus effuses* L.	[42]
Juncuenins G (**42**)	IC_50_ (MCF-7) > 100 µM; IC_50_ (HeLa) > 100 µM.	*Juncus effuses* L.	[42]
4,7-dihydroxy-2-methoxy-9,10-dihydrophenanthrene (**43**)	IC_50_ (MCF-7) = 9.17 µM; IC_50_ (HeLa) = 19.6 µM.	*Juncus effuses* L.	[42]
Lusianthridin (**44**)	IC_50_ (A549) = 7.7 µM; IC_50_ (SK-OV-3) = 37.1 ± 2.8 µM; IC_50_ (KCR) = 35.8 ± 1.7 µM	*Dendrobium nobile Lindl.*	[44]
Orchinol (**45**)	IC_50_ (HI-60) = 11.96 µM; IC_50_ (THP-1) = 8.92 µM.	*Dendrobium officinale Kimura & Migo*	[13]
Spiranthesphenanthrine A (**46**)	IC_50_ (B16-F10) = 19.0 ± 7.3 µM.	*Orchid plant Panlongshen*	[45]
9,10-dihydro-4,7-dimethoxyphenanthrene-2,8-diol (**47**)	IC_50_ (RAW) = 25.0 to 87.2 µM.	*Bai Ji*	[46]
2,7-dihydroxy-4,6-dimethoxyphenylene (**48**)	IC_50_ (786-0) = 56.98 ± 9.29 µM; IC_50_ (MCF-7) = 46.99 ± 5.55 µM; IC_50_ (Hep2) > 100 µM; IC_50_ (UACC-62) = 2.59 ± 0.11 µM; IC_50_ (NCI/ADR-RES) = 58.83 ± 2.33 µM.	*Combretum laxum*	[47]
2,6-dihydroxy-3,4,7-trimethoxy-9,10-dihydrophenanthrene (**49**)	IC_50_ (786-0) > 100 µM; IC_50_ (MCF-7) = 42.01 ± 9.33 µM; IC_50_ (Hep2) > 100 µM; IC_50_ (UACC-62) > 100 µM; IC_50_ (NCI/ADR-RES) > 100 µM.	*Combretum laxum*	[47]
2,6-dihydroxy-4,7-dimethoxy-9,10- dihydrophenanthrene (**50**)	IC_50_ (786-0) > 100 µM; IC_50_ (MCF-7) > 100 µM; IC_50_ (Hep2) = 47.58 ± 0.11µM; IC_50_ (UACC-62) = >100 µM; IC_50_ (NCI/ADR-RES) > 100 µM.	*Combretum laxum*	[47]
Phytol (**51**)	IC_50_ (P388D_1_) = 75.0 µM.	*Pholidota cantonensis Rolfe*	[48]
Phocantone (**52**)	IC_50_ (P388D_1_) = 27.5 µM.	*Pholidota cantonensis Rolfe*	[48]
Shancidin (**53**)	IC_50_ (SMMC-7721) = 12.57 µM; IC_50_ (A549) = 18.21 µM; IC_50_ (MGC80-3) = 11.6 µM	*Cymbidium hybridum*	[49,50]
9,9′,10,10′-tetrahydro-3,3′-biphenanthrene (**54**)	IC_50_ (SGC-7901) = 63.8 ± 3.6 µM; IC_50_ (HepG2) = 78.4 ± 29.0 µM; IC_50_ (KCR) = 58.2 ± 2.6 µM	*Orchid plant Panlongshen*	[45]
**56**	IC_50_ (HeLa) = 25 µM; IC_50_ (MCF-7) = 31 µM; IC_50_ (A431) = 42 µM	*/*	[51]
Compressin B (**68**)	IC_50_ (HeLa) = 1.86 µM.	*J. compressus*	[57]
Dendrocandin P1 (**69**)	IC_50_ (HI-60) = 35.32 ± 1.76 µM; IC_50_ (THP-1) = 20.78 ± 1.80 µM.	*Dendrobium officinale stem*	[13,58,59,60,61,62,63]
Dendrocandin P2 (**70**)	IC_50_ (HI-60) > 50 µM; IC_50_ (THP-1) = 45.32 ± 2.39 µM.	*Dendrobium officinale stem*	[13,58,59,60,61,62,63]
Denbinobin (**71**)	IC_50_ (MCF-7) = 13.13 ± 0.47 µM; IC_50_ (HL-60) = 3.08 ± 0.12 µM; IC_50_ (A549) = 19.68 ± 1.12 µM; IC_50_ (SW480) = 16.81 ± 0.13 µM.	*D. candidum*; *D. nobile*; *D. venustum*	[64,65,66,67,68,73]
Ephemeranthoquinone (**72**)	IC_50_ (MCF-7) = 3.63 ± 0.03 µM; IC_50_ (HL-60) = 2.33 ± 0.12 µM; IC_50_ (A549) = 14.97 ± 0.64 µM; IC_50_ (SW480) = 6.66 ± 0.71 µM.	*D. hancockii*, *D. hongdie*; *D. longicornu*; *D. plicatile*	[69,70,71,72,73]
8-methoxy-12-(4-methoxybenzyl)-13,14-dihydro-12*H*-naphtho [2,1-a]xanthene- 2,5,9,10-tetraol (**74**)	IC_50_ (MDA-231) = 25.2 µM; IC_50_ (HepG2) = 51.3 µM; IC_50_ (HT-29) = 30.4 µM.	*Dendrobium officinale*	[76]
Erathrin A (**75**)	IC_50_ (HeLa) = 14.5 µM.	*Wild peony*	[77]
3′,7′,7-trihydroxy-2,2′,4′-trimethoxy-[1,8′-biphenanthrene]-3,4-dione (**76**)	IC_50_ (MCF-7) = 12.6 µM; IC_50_ (HT-29) = 22.7 µM; IC_50_ (HUVEC) = 33.5 µM; IC_50_ (A549) = 22.6 µM.	*Bai ji*	[78]
**77**	IC_50_ (DU145) = 1.5 ± 0.09 µM; IC_50_ (HeLa) = 2.9 ± 0.19 µM.	*/*	[79]
Bleochranol A (**78**)	IC_50_ (HL-60) = 0.24 ± 0.03 µM; IC_50_ (SMMC-7721) = 12.22 ± 0.26 µM; IC_50_ (A549) = 3.51 ± 0.09 µM; IC_50_ (MCF-7) = 3.33 ± 0.09 µM; IC_50_ (SW480) = 12.97 ± 0.34 µM.	*Bletilla striata*	[80]
Bleochranol B (**79**)	IC_50_ (HL-60) => 40 µM; IC_50_ (SMMC-7721) > 40 µM; IC_50_ (A549) = 34.87 ± 0.40 µM; IC_50_ (MCF-7) = 29.07 ± 1.34 µM; IC_50_ (SW480) > 40 µM.	*Bletilla striata*	[80]
Bleochranol C (**80**)	IC_50_ (HL-60) = 15.05 ± 0.33 µM; IC_50_ (SMMC-7721) = 19.85 ± 0.42 µM; IC_50_ (A549) = 19.16 ± 0.41 µM; IC_50_ (MCF-7) = 18.84 ± 0.41 µM; IC_50_ (SW480) = 18.61 ± 0.68 µM.	*Bletilla striata*	[80]
Bleochranol D (**81**)	IC_50_ (HL-60) = 10.65 ± 0.09 µM; IC_50_ (SMMC-7721) = 17.95 ± 0.44 µM; IC_50_ (A549) = 18.32 ± 0.44 µM; IC_50_ (MCF-7) = 17.62 ± 0.81 µM; IC_50_ (SW480) = 18.60 ± 0.99 µM.	*Bletilla striata*	[80]
Juncunol (**82**)	IC_50_ (HepG2) = 18 µM.	*Juncus effuses* L.	[81]
AL-BII (**83**)	IC_50_ (HepG2) = 0.2 µM.	*Aristolochia*; *Asarum*; *Akebia*; *Clematis*, *Stephania*; *Menispermum*; *Dauricum*; *Asteraceae*	[82,84]
AAI (**84**)	IC_50_ (HepG2) = 9.7 µM.	*Clematis*, *Stephania*; *Menispermum*; *Dauricum*; *Asteraceae*	[82,84]

## Data Availability

No new data were created or analyzed in this study. Data sharing is not applicable to this article.
